# TLR2 Signaling Contributes to Rapid Inflammasome Activation during *F. novicida* Infection

**DOI:** 10.1371/journal.pone.0020609

**Published:** 2011-06-16

**Authors:** Crystal L. Jones, David S. Weiss

**Affiliations:** 1 Department of Microbiology and Immunology, Emory University School of Medicine, Atlanta, Georgia, United States of America; 2 Emory Vaccine Center, Emory University School of Medicine, Atlanta, Georgia, United States of America; 3 Division of Infectious Diseases, Department of Medicine, Emory University School of Medicine, Atlanta, Georgia, United States of America; Universidad Nacional, Heredia, Costa Rica

## Abstract

**Background:**

Early detection of microorganisms by the innate immune system is provided by surface-expressed and endosomal pattern recognition receptors (PRRs) such as Toll-like receptors (TLRs). Detection of microbial components by TLRs initiates a signaling cascade leading to the expression of proinflammatory cytokines including IL-6 and IL-1β. Some intracellular bacteria subvert the TLR response by rapidly escaping the phagosome and entering the cytosol. However, these bacteria may be recognized by the inflammasome, a multi-protein complex comprised of a sensor protein, ASC and the cysteine protease caspase-1. Inflammasome activation leads to release of the proinflammatory cytokines IL-1β and IL-18 and death of the infected cell, an important host defense that eliminates the pathogen's replicative niche. While TLRs and inflammasomes are critical for controlling bacterial infections, it is unknown whether these distinct host pathways cooperate to activate defenses against intracellular bacteria.

**Methodology/Significant Findings:**

Using the intracellular bacterium *Francisella novicida* as a model, we show that TLR2^−/−^ macrophages exhibited delayed inflammasome activation compared to wild-type macrophages as measured by inflammasome assembly, caspase-1 activation, cell death and IL-18 release. TLR2 also contributed to inflammasome activation in response to infection by the cytosolic bacterium *Listeria monocytogenes*. Components of the TLR2 signaling pathway, MyD88 and NF-κB, were required for rapid inflammasome activation. Furthermore, TLR2^−/−^ mice exhibited lower levels of cell death, caspase-1 activation, and IL-18 production than wild-type mice upon *F*. *novicida* infection.

**Conclusions/Significance:**

These results show that TLR2 is required for rapid inflammasome activation in response to infection by cytosolic bacterial pathogens. In addition to further characterizing the role of TLR2 in host defense, these findings broaden our understanding of how the host integrates signals from spatiotemporally separated PRRs to coordinate an innate response against intracellular bacteria.

## Introduction

The mammalian innate immune system defends against a variety of microbial pathogens. Early detection of invading microorganisms is provided by germline-encoded pattern recognition receptors (PRRs) that recognize conserved microbial components known as pathogen-associated molecular patterns (PAMPs). The macrophage, which acts as a sentinel of the innate immune system, is equipped with numerous membrane bound and cytosolic PRRs that can detect microbes. Recognition of PAMPs by PRRs enables the macrophage to rapidly mount a proinflammatory response aimed at eliminating microorganisms. One of the best-characterized families of PRRs is the Toll-like receptors (TLRs). TLRs are type I integral membrane proteins that recognize PAMPs such as lipopolysaccharide (TLR4), bacterial lipoproteins (TLR2), flagellin (TLR5), and CpG DNA (TLR9) [1]. TLRs detect these foreign molecules at the plasma membrane and within phagosomes, resulting in the initiation of signaling cascades that lead to the activation of the transcriptional regulator NF-κB and the expression of proteins involved in host defense including the proinflammatory cytokines IL-6, TNF-α, and IL-1β [1].

Some bacterial pathogens evade TLRs by physically escaping the phagosome and reaching the cytosol where they replicate [2]. In order to detect and defend against these pathogens, the host uses cytosolic sensors such as the Nod-like receptor (NLR) and AIM2-like receptor (ALR) families. NLRs, such as NLRP3, respond to a diverse set of stimuli [3] while ALRs can bind to double-stranded DNA released from viruses and bacteria [4]. Detection of PAMPs by either NLRs or AIM2 activates the inflammasome, a multiprotein complex composed of a sensor protein (NLR/AIM2), the adaptor protein ASC, and the cysteine protease caspase-1 [5]. Inflammasome activation leads to death of the infected macrophage, which is a host defense response that is thought to be protective since it eliminates the pathogen's replicative niche [6]. In addition to inducing host cell death, inflammasome activation also leads to the caspase-1-mediated processing and maturation of the pro-forms of the proinflammatory cytokines IL-1β and IL-18 and their release from dying cells.

While both TLRs and the inflammasome can be activated during infection, if and how these host defense systems cooperate during infection remains unclear. However, these host defenses have been shown to work together under other circumstances. For example, the NLRP3 inflammasome is activated by a two-step process, which is initiated by priming macrophages with LPS or other TLR agonists to induce the expression of NLRP3 [7]. This priming step is also required to induce the expression of pro-IL-1β, whereas expression of pro-IL-18 is constitutive in macrophages [8]. Enhanced NLRP3 expression is required for responsiveness to the second stimulus, ATP, which signals through the P2X_7_ receptor (P2X_7_R) [9] and induces inflammasome activation, leading to cell death and the release of both IL-1β and IL-18. While this suggests that TLRs can act to help facilitate NLRP3 inflammasome activation under certain conditions, it is unclear if this type of cooperation occurs during the activation of inflammasomes in the context of a bacterial infection.

*Francisella novicida* is a Gram-negative intracellular bacterium that has been used as a model organism to study various aspects of inflammasome activation [10]. Shortly after being internalized by a macrophage, this bacterium escapes the phagosome and begins to replicate in the cytosol where it is recognized by the AIM2 inflammasome [11], [12], but not the NLRP3 [9] or NLRC4 inflammasomes [13]. *F. novicida* is closely related to the highly virulent *F. tularensis*, which causes the disease tularemia in humans and has been categorized as a potential bioweapon by the Centers for Disease Control and Prevention [14]. Additionally, *F. novicida* causes a tularemia-like disease in mice, allowing for the study of the role of the inflammasome in host defense during *in vivo* infection [15].

Although *Francisella* species are capable of invading an array of cells during infection [16], macrophages are considered to be the first cells that the bacteria encounter, and this interaction is critical for establishment of disease. TLR2, which signals in response to *Francisella* lipoproteins [17], is the primary TLR involved in recognizing and responding to this pathogen. *Francisella* LPS contains modifications that abrogate TLR4 recognition [18], the bacterium lacks genes that encode flagella [19] and therefore does not activate TLR5 [20], [21], and TLR9 plays a minor role, if any, in providing the host with protection against infection *in vivo* [22]. Furthermore, TLR2 and the TLR adaptor protein MyD88 are critical for host defense against *Francisella* infection since mice lacking these proteins are more susceptible to infection than their wild-type counterparts [22]–[24]. After *F. novicida* escapes the phagosome and replicates in the cytosol of macrophages, it is recognized by the AIM2 inflammasome complex which is essential for controlling *F. novicida* infection *in vivo* since mice lacking AIM2, ASC, or caspase-1 are highly susceptible to infection [11], [13].

Due to the sequential activation of TLR2 and the AIM2 inflammasome during *F*. *novicida* infection of macrophages and the critical role that both of these recognition systems play in host defense against this bacterium, we tested whether these systems cooperate during infection. We found that TLR2 contributes to the rapid induction of inflammasome assembly, caspase-1 activation, host cell death and IL-18 release, since TLR2^−/−^ macrophages have a significant delay in these inflammasome-dependent responses during *F. novicida* infection. Furthermore, TLR2 contributes to rapid inflammasome activation by signaling through MyD88 and NF-κB in infected macrophages. These findings were further validated with *in vivo* experiments showing that TLR2 contributes to inflammasome activation during *F. novicida* infection. Together these results highlight a novel way in which TLR2 contributes to innate immune signaling during bacterial infection. Furthermore, these findings demonstrate that innate recognition systems localized in different macrophage compartments that are activated at different times during infection, can cooperate to provide a multi-tiered defense response against intracellular bacterial pathogens.

## Materials and Methods

### Ethics Statement

All experimental procedures were approved by the Emory University Institutional Animal Care and Use Committee (protocol #069-2008Y).

### Bacterial strains and growth conditions

*Francisella novicida* strain U112 and an isogenic *F. novicida mglA* mutant previously described [25] were obtained from Dr. Denise Monack (Stanford University, Stanford, CA). *E. coli* DH5α was obtained from Invitrogen (Carlsbad, CA). All bacterial cultures were grown overnight at 37°C with aeration in tryptic soy broth (BD Biosciences, Sparks, MD), and *F. novicida* cultures were supplemented with 0.2% L-cysteine (BD Biosciences). Bacteria were killed by incubating in a water bath heated to 100°C for 30 minutes. Heat-killed bacteria were plated on tryptic soy agar (TSA) supplemented with 0.1% L-cysteine to ensure that all bacteria were killed after treatment.

### Mice

Wild-type and P2X_7_R^−/−^ C57BL/6J mice were obtained from Jackson Laboratories (Bar Harbor, ME). TLR2^−/−^ and MyD88^−/−^ C57BL/6 mice were generous gifts from Dr. Bali Pulendran (Emory Vaccine Center, Atlanta, GA). All animals were housed under specific pathogen-free conditions in filter-top cages at the Emory Vaccine Center vivarium and provided with sterile water and food *ad libitum*. All animal studies were reviewed and approved by the Emory University Institutional Animal Care and Use Committee.

### Macrophage experiments and infections

Murine bone marrow-derived macrophages (BMDM) were prepared from wild-type, TLR2^−/−^, MyD88^−/−^ and P2X_7_R^−/−^ C57BL/6 mice and cultured as described [26]. Macrophages were cultured in 96-well plates (8×10^4^ cells/well) or 6-well plates (1.8×10^6^ cells/well) in high glucose Dulbecco's modified Eagle's medium (DMEM) (Lonza, Walkersville, MD) supplemented with 10% heat-inactivated fetal bovine serum (HyClone, Logan, UT) and 10% L929-conditioned media (conditioned DMEM) overnight. The media was removed and bacteria were added at the indicated multiplicities of infection (MOI) expressed as bacteria per macrophage. Plates were spun for 15 minutes at 2,000 rpm at room temperature to promote uptake of bacteria. Macrophages were incubated for 30 minutes at 37°C and washed two times before adding warm conditioned DMEM. To assess TLR signaling, macrophages were treated with Pam_3_CSK_4_ (100 µg/ml) (Invivogen, San Diego, CA), UltraPure LPS (100 µg/ml) (Invivogen), heat-killed *F. novicida* (100 killed bacteria/macrophage) and supernatants were collected after 6 h. The concentration of IL-6 in culture supernatants was quantified by ELISA (BD Biosciences). To inhibit NF-κB, macrophages were treated with 20 µM of caffeic acid phenethyl ester (CAPE) (EMD, Gibbstown, NJ) for 2 hours prior to infection [27], [28]. For cell death assays, culture supernatants were collected at the specified timepoints after infection and cell death was quantified colorimetrically using the Cyto-Tox96 lactate dehydrogenase (LDH) release kit according to the manufacturer's instructions (Promega, Madison, WI). IL-18 (MBL International Corporation, Woburn, MA) and IL-1β (BD Biosciences) levels in the supernatants were quantified by ELISA.

### Immunoblotting

Macrophages were infected with bacteria at a multiplicity of infection of 100∶1 and lysed with Lysis Buffer (2 mM DTT and 10% NP-40) supplemented with Complete Protease Inhibitor Cocktail (Roche, Indianapolis, IN). Proteins were resolved by SDS-PAGE using 4–15% polyacrylamide mini-gels (Bio-Rad, Hercules, CA) and transferred to nitrocellulose membranes. Blots were probed with rat anti-mouse caspase-1 p20 clone 4B4.2.1, rat anti-mouse ASC clone 8E4.1 (both generous gifts from Dr. Sanjeev Mariathasan, Genentech, South San Francisco, CA) and anti-mouse β-actin (Sigma-Aldrich, St. Louis, MO) antibodies. Proteins were visualized by SuperSignal ECL substrate (Thermo Scientific, Rockford, IL) and detected using the UVP Multispectral Imaging System (Upland, CA).

### Macrophage staining and immunofluorescence

Macrophages were seeded onto glass cover slips in 24-well plates (3×10^5^ cells/well) and infected as described above. Infected cells were washed and fixed with 4% paraformaldehyde for 10 minutes at 37°C. Cells were incubated with primary antibodies against ASC (clone 8E4.1) and the p10 subunit of caspase-1 (Santa Cruz Biotechnology Inc, Santa Cruz, CA) for 30 minutes at 37°C. Following incubation, cells were washed three times with PBS and incubated with the appropriate Alexa Fluor–conjugated secondary antibodies and phalloidin stain (Invitrogen) for 30 minutes at 37°C. Cells were washed three times and cover slips were mounted over SlowFade Gold antifade reagent containing DAPI (Invitrogen). Images were obtained using a Zeiss Axioscope Z.1 microscope equipped with a Zeiss Imager 2.1 camera, and images were taken at 40× magnification. AxioVision software 4.6.3 was used for image acquisition. Speck formation was quantified by measuring the percentage of macrophages containing ASC or ASC-caspase-1 specks in a total of 10 fields for each sample.

To measure phagosomal escape, macrophages were pre-chilled at 4°C and infected with *F. novicida* at an MOI of 100∶1. Cells were then rapidly warmed for 5 min in a 37°C water bath to allow for bacterial uptake. Macrophages were incubated for 10 min at 37°C, then washed three times with warm DMEM to remove extracellular bacteria, and incubated at 37°C until the indicated timepoints. At 30 and 60 minutes post-infection, cells were washed three times with PBS and fixed with 4% paraformaldehyde for 10 minutes at 37°C. Following fixation, cells were incubated with primary antibodies against LAMP-1 (1D4B) (Developmental Studies Hybridoma Bank, University of Iowa, Iowa City, IA) and *F. novicida* (generous gift from Dr. Denise Monack, Stanford University, Stanford, CA) for 30 minutes at 37°C. Following incubation, cells were washed three times with PBS and incubated with the appropriate Alexa Fluor–conjugated secondary antibodies and phalloidin stain (Invitrogen) for 30 minutes at 37°C. Cover slips were mounted over SlowFade Gold antifade reagent containing DAPI. Images were obtained using a Zeiss Axioscope Z.1 microscope equipped with a Zeiss Imager 2.1 camera, and images were taken at 63× magnification. To visualize and enumerate bacteria colocalizing with LAMP-1, Volocity software 5.5 (Improvision) was used to assemble z-stacks into 3D images.

### Mouse infections

Groups of five wild-type and TLR2^−/−^ mice were infected intraperitoneally with 2×10^6^ CFU of *F. novicida* suspended in 500 µL of sterile PBS. At 4 hours post-infection, blood was collected from the mice and serum was prepared. The livers of infected mice were harvested and divided. One half of a lobe of the liver was homogenized and dilutions were plated on Mueller-Hinton agar supplemented with 0.1% L-cysteine for enumeration of bacterial levels in each organ. The other half was frozen in O.C.T. compound (TissueTek, Torrance, CA) and stored at −80°C until being sectioned.

### Tissue staining and immunofluorescence

Frozen livers from infected mice were cut into 8 µm sections. To visualize cell death, liver sections were stained using a fluorescent In Situ Cell Death Detection Kit (Roche) according to the manufacturer's instructions. Bacteria were labeled using a chicken anti-*F. novicida* antibody (generous gift from Dr. Denise Monack, Stanford University, Stanford, CA). Coverslips were mounted over SlowFade Gold antifade reagent containing DAPI (Invitrogen). The number of TUNEL positive cells per field was calculated in a total of 10 fields for each sample. For detection of active caspase-1, liver sections were stained with goat anti-caspase-1 p20 antibody (Santa Cruz Biotechnology Inc, Santa Cruz, CA) and donkey anti-goat IgG-FITC (Santa Cruz Biotechnology, Inc). Mature caspase-1 p20 was quantified using single channel densitometry to measure the total fluorescence emission of FITC-labeled liver sections. Images were obtained using a Zeiss Axioscope Z.1 microscope equipped with a Zeiss Imager 2.1 camera, and images were taken at 20× magnification. AxioVision software 4.6.3 was used for image acquisition and densitometry measurements.

### Statistical analysis

The statistical significance of all data in this manuscript was analyzed using the Student's t-test or Mann-Whitney test with GraphPad software (GraphPad, La Jolla, CA).

## Results

### TLR2 contributes to rapid inflammasome activation during *F. novicida* infection

TLR signaling has been shown to act in synergy with cytosolic host defense systems to elicit a proinflammatory response against bacteria [7], [29]–[32]. Considering these findings and the fact that *F. novicida* activates TLR2 and the cytosolic AIM2 inflammasome, we investigated whether TLR2 contributes to inflammasome activation during *F. novicida* infection. While LPS-prestimulated macrophages are often used to study inflammasome activation, we used unstimulated macrophages in our experiments for several reasons: 1) to examine the role of TLR2 during natural infection in the absence of exogenous stimuli, 2) to mimic the early events of natural infection, and 3) because *F. novicida* makes an LPS with very low stimulatory activity [18] and TLR4 is not normally activated during infection with *Francisella*.

**Figure 1:**
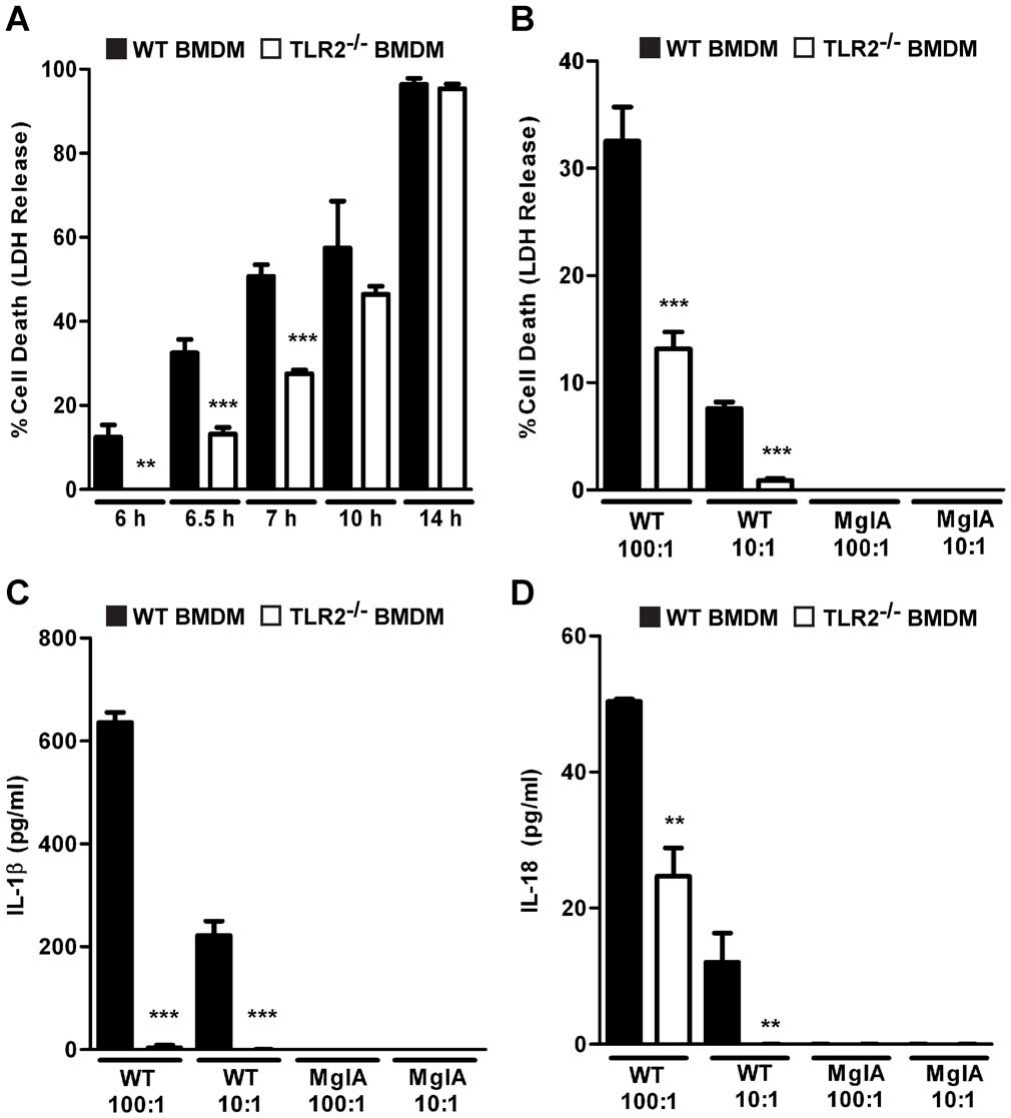
TLR2 is required for rapid inflammasome activation in response to *F. novicida* infection. (A) Bone marrow-derived macrophages (BMDM) from wild-type and TLR2^−/−^ C57BL/6 mice were infected with wild-type *F. novicida* at an MOI of 100∶1 and supernatants were collected at 6–14 h post-infection for quantification of macrophage death as measured by LDH release. (B) Macrophages were infected with wild-type (WT) and *mglA* mutant strains of *F*. *novicida* (MglA) at the indicated MOIs for 6.5 h and supernatants were collected for quantification of cell death, and levels of (C) IL-1β and (D) IL-18. Data are representative of five independent experiments. Error bars represent the standard deviation of triplicate samples. *** p≤0.0007, ** p<0.0085.

The hallmarks of inflammasome activation are host cell death, proteolytic maturation of caspase-1, and the secretion of the proinflammatory cytokines IL-1β and IL-18 [5]. We first assayed for host cell death by quantifying the level of lactate dehydrogenase (LDH) released into the supernatants of infected macrophages after infection. At 6 h post-infection, soon after *F. novicida* starts to replicate in the cytosol [33], 12% of wild-type macrophages were dead while no death was detectable in TLR2^−/−^ cells (Figure 1A). The delayed cell death phenotype was observed in TLR2^−/−^ macrophages up to 10 h post-infection. This delay in cell death was observed at MOIs of 10∶1 and 100∶1 (Figure 1B). By 14 h post-infection, the levels of cell death in wild-type and TLR2^−/−^ macrophages were equivalent (∼96%) (Figure 1A), demonstrating that TLR2 contributes to rapid inflammasome-dependent cell death but is not absolutely required for this process. This is in contrast to macrophages from ASC^−/−^ and caspase-1^−/−^ mice, which did not undergo cell death between 6 and 14 h post-infection (data not shown), in agreement with previous studies [13]. The rapid cell death response was dependent on escape of the bacteria into the cytosol since infection with an *F. novicida* strain unable to escape the phagosome (due to a point mutation in the *mglA* gene, which encodes a transcriptional regulator required for phagosomal escape) did not induce host cell death (Figure 1B). This is also in agreement with previous studies [13].

To ensure that the delayed induction of cell death exhibited by TLR2^−/−^ macrophages was not due to an inability of *F. novicida* to reach and replicate in the cytosol of these cells, we measured phagosomal escape and intracellular replication. Between 30 and 60 minutes post-infection, there was a decrease in the number of LAMP-1 associated bacteria (Figure S1A, and in agreement with kinetics observed previously [26], [34]), which is indicative of bacteria escaping the phagosome. Since phagosomal escape is required for *Francisella* to replicate in macrophages [25], [35], we measured the intracellular replication of the bacteria and found that they were able to replicate with similar kinetics in both wild-type and TLR2^−/−^ macrophages (Figure S1B), also consistent with previous studies [24], [36]. Taken together, these results demonstrate that the role of TLR2 in promoting rapid inflammasome activation during *F. novicida* infection cannot be explained by differences in bacterial trafficking or replication.

To further test the role of TLR2 in inflammasome-dependent responses, we measured the levels of IL-1β and IL-18 in macrophage supernatants following infection with *F. novicida*. IL-1β secretion is often used as a marker for inflammasome activation, but TLR2 regulates the expression of pro-IL-1β in response to *Francisella* infection, complicating the use of this cytokine as a marker for inflammasome activity [37]. As expected, supernatants collected from TLR2^−/−^ macrophages infected with *F. novicida* for 6.5 h at an MOI of 100∶1 were devoid of IL-1β, whereas wild-type macrophages secreted IL-1β (Figure 1C). Since we cannot separate the role of TLR2 in transcriptionally controlling pro-IL-1β expression from an effect on inflammasome activation, we did not use IL-1β as a marker in this study. Instead we used IL-18 which is constitutively transcribed and expressed at the protein level independently of TLR2 (Figure S2A, B) [8]. At 6.5 h post-infection, the supernatants of wild-type macrophages infected with *F. novicida* contained significantly increased levels of IL-18 compared to infected TLR2^−/−^ macrophages (Figure 1D). Furthermore, IL-18 release was also dependent on bacterial escape into the cytosol since the *mglA* mutant did not induce this response. The role of TLR2 in promoting IL-18 secretion further demonstrates that TLR2 plays a role in the rapid induction of the inflammasome during *F. novicida* infection.

### TLR2 contributes to rapid inflammasome assembly and caspase-1 processing in response to *F. novicida* infection

**Figure 2:**
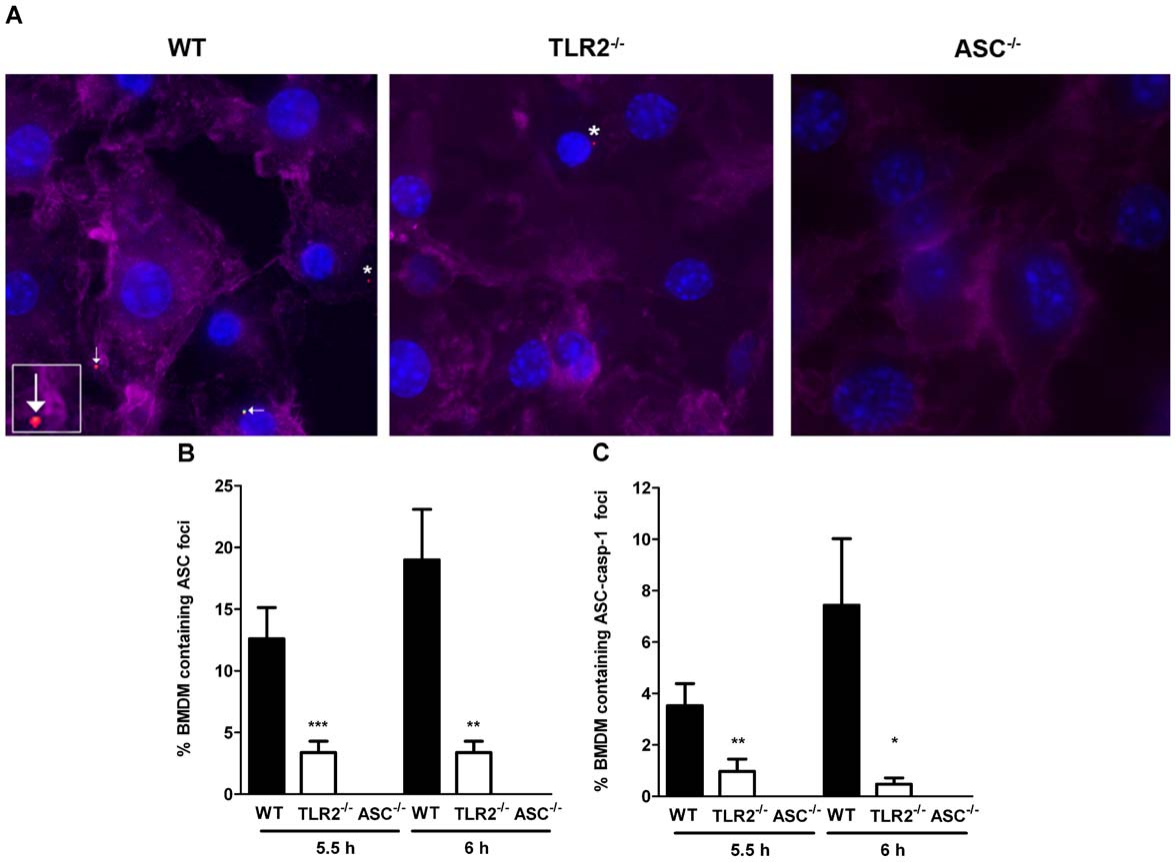
TLR2 contributes to rapid inflammasome assembly during infection with *F. novicida*. Wild-type and TLR2^−/−^ BMDM were infected with *F*. *novicida* at an MOI of 100∶1 for 6 h. (A) Macrophages were stained for the presence of caspase-1 (green), ASC (red), DNA (blue) and actin (purple) and visualized by fluorescence microscopy. Stars show ASC foci and arrows point to ASC and caspase-1 colocalized foci. Inflammasome assembly was quantified by counting the number of macrophages containing (B) ASC foci or (C) ASC-caspase-1 colocalized foci. Error bars represent the standard error of the mean (SEM). Data are representative of two independent experiments, in which at least 200 macrophages were counted for each sample. *** p = 0.0008, ** p <0.0040, * p = 0.0167.

Since inflammasome activity is dependent upon assembly of this complex [38], we investigated whether the delay in host cell death and IL-18 release exhibited by TLR2^−/−^ macrophages during *F. novicida* infection was due to a delay in complex formation. Inflammasome complexes are formed when ASC is recruited from a diffuse localization in the cytosol to a single cytosolic focus, to which procaspase-1 is recruited [39]. Using fluorescence microscopy to visualize inflammasome complex formation, we found that TLR2^−/−^ macrophages exhibited delayed ASC foci formation compared with wild-type macrophages during *F. novicida* infection (Figure 2A). At 5.5 h post-infection, before any detectable cell death had occurred, 12% of wild-type macrophages infected with *F. novicida* contained ASC foci while only 3% of infected TLR2^−/−^ macrophages contained ASC foci (Figure 2B). Interestingly, TLR2^−/−^ macrophages also contained fewer ASC foci colocalizing with caspase-1, compared to wild-type macrophages (Figure 2C). This defect was still apparent after macrophages began to undergo cell death at 6 h post-infection (Figure 2B, C and Figure 1A). To ensure that the inflammasome complexes formed in response to *F. novicida* infection were dependent on ASC, we infected ASC^−/−^ macrophages with *F. novicida* and found that these macrophages were unable to form ASC or caspase-1 foci (Figure 2A-C). These results demonstrate that TLR2 promotes rapid inflammasome assembly in response to *F. novicida* infection.

**Figure 3:**
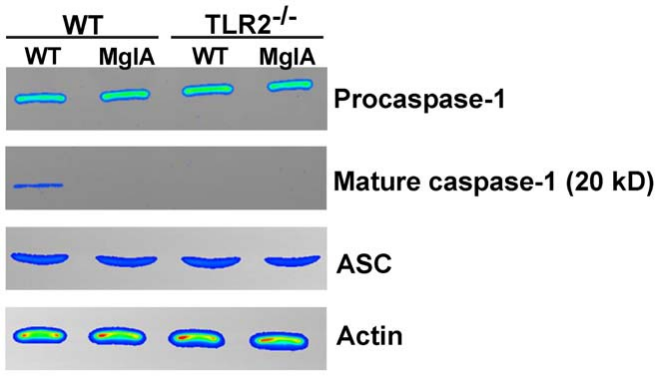
TLR2 is required for rapid activation of caspase-1 during *F. novicida* infection. BMDM were infected with wild-type (WT) and *mglA* mutant strains of *F*. *novicida* (MglA) at an MOI of 100∶1 and lysed at 6 h post-infection. The levels of procaspase-1, mature caspase-1 (p20 subunit) and ASC in infected macrophages were measured by Western blot. β-actin was used as a loading control. Data are representative of three independent experiments.

Proteolytic maturation of caspase-1 is dependent on inflammasome assembly and required for inflammasome activity [38], [40], [41]. Therefore, we measured the levels of processed caspase-1 in wild-type and TLR2^−/−^ macrophages following *F. novicida* infection. At 6 h post-infection, we detected the processed p20 subunit of caspase-1 in lysates from infected wild-type, but not TLR2^−/−^ macrophages (Figure 3). We could detect processed caspase-1 at 7.5 h post-infection in TLR2^−/−^ macrophages, but the level was still less than that observed in wild-type macrophages (Figure S3). This difference in caspase-1 maturation was independent of any differences in procaspase-1 levels, which were equivalent in wild-type and TLR2^−/−^ macrophages (Figure 3). Furthermore, the expression of the other known inflammasome components ASC (Figure 3) and AIM2 (Figure S4) were also equal in infected wild-type and TLR2^−/−^ macrophages, demonstrating that induction of the expression of inflammasome components does not explain the role of TLR2 in promoting inflammasome activity. In agreement with the data from Figure 1, bacterial escape into the cytosol was essential for the induction of caspase-1 processing since the *mglA* mutant did not induce this response (Figure 3). Taken together, these data demonstrate that TLR2 promotes rapid inflammasome assembly during *F. novicida* infection of macrophages, leading to more rapid caspase-1-processing and inflammasome activation.

### Components of the TLR2 signaling pathway are required for rapid inflammasome activation

Since we have shown that TLR2 signaling contributes to rapid *F. novicida*-induced inflammasome activation, we examined whether TLR2 signaling alone was sufficient to induce this response. Macrophages treated with the TLR2 agonist Pam_3_CSK_4_ or heat-killed *F. novicida*, which contains bacterial lipoproteins (BLPs) that activate TLR2, did not induce any host cell death, suggesting that TLR2 signaling alone is not sufficient for this response (Figure S5). In addition, macrophages infected with the *mglA* mutant strain of *F. novicida*, as a control, were unable to induce host cell death. To demonstrate the TLR2-activating capacity of these stimuli, we measured the levels of IL-6 in the supernatants of stimulated wild-type and TLR2^−/−^ macrophages. All of the stimuli induced IL-6, confirming that our preparations were active (Figure S5A). As expected, Pam_3_CSK_4_, heat-killed *F. novicida*, and the *mglA* mutant all induced IL-6 in a strictly TLR2-dependent manner. As a control for the specificity of the TLR2^−/−^ macrophages, we demonstrated that LPS, a TLR4 agonist, induced IL-6 secretion in both wild-type and TLR2^−/−^ macrophages. Collectively, and in agreement with previous results [13], [42], these data show that TLR2 signaling alone is not sufficient to induce inflammasome activation.

**Figure 4:**
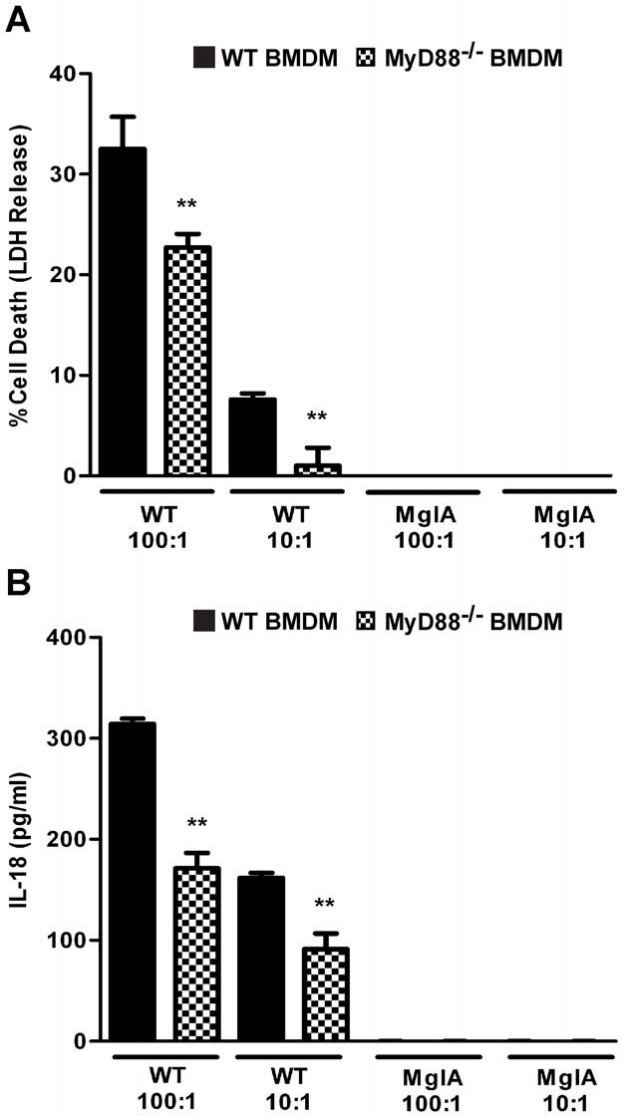
MyD88 is involved in rapid inflammasome activation in response to *F. novicida* infection. BMDM from wild-type and MyD88^−/−^ C57BL/6 mice were infected with wild-type (WT) and *mglA* mutant strains of *F*. *novicida* (MglA) at the indicated MOIs. At 6.5 h post-infection, (A) death of the infected macrophages was measured by LDH release and (B) the concentration of IL-18 in the supernatants was quantified by ELISA. Data are representative of four independent experiments. Error bars represent the standard deviation of triplicate samples. ** p<0.0090.

**Figure 5:**
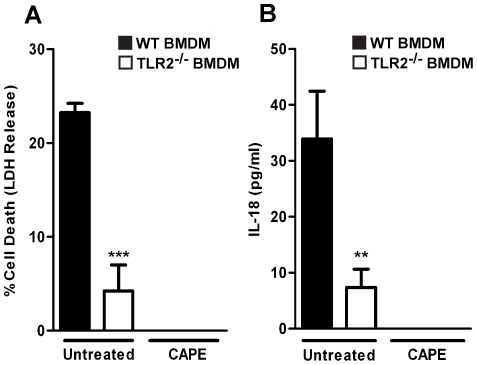
NF-κB activation contributes to rapid inflammasome activation during *F. novicida* infection. Macrophages were left untreated or treated with 20 µM of CAPE (NF-κB inhibitor) for 2 h, then infected with *F. novicida* at an MOI of 100∶1. Supernatants were collected for measurement of cell death by quantifying LDH release (A) and IL-18 secretion (B) at 6.5 h post-infection. Data are representative of four independent experiments. Error bars represent the standard deviation of triplicate samples. *** p≤0.0004, ** p = 0.0074.

Next, we sought to determine if known components of the TLR2 signaling pathway contribute to rapid inflammasome activation in response to *F. novicida* infection. The TLR2 adaptor protein MyD88 is absolutely required for *Francisella*-induced cytokine production in macrophages [36], and we therefore investigated if it also played a role in rapid inflammasome activation. MyD88^−/−^ macrophages infected with wild-type *F. novicida* exhibited significantly less cell death (Figure 4A) and secreted less IL-18 (Figure 4B) than wild-type macrophages, showing that MyD88 is involved in rapid inflammasome activation. The rapid cell death response was also dependent on phagosomal escape since infection with the *mglA* mutant strain of *F. novicida* did not induce host cell death (Figure 4A). This difference was not due to an inability of *F. novicida* to replicate in MyD88^−/−^ macrophages (Figure S1). To determine whether NF-κB-dependent signaling played a role in rapid activation of the inflammasome during *F. novicida* infection, we pre-treated macrophages with caffeic acid phenethyl ester (CAPE), which has previously been shown to block NF-κB translocation to the nucleus of macrophages infected with *F. novicida* [27], [28]. CAPE-treated wild-type and TLR2^−/−^ macrophages did not undergo cell death (Figure 5A) or secrete IL-18 (Figure 5B) after *F. novicida* infection. These results demonstrate that the TLR2 signaling components MyD88 and NF-κB play a role in inducing rapid inflammasome activation during *F. novicida* infection.

### *F. novicida*-induced inflammasome activation is independent of the P2X_7_R pathway

**Figure 6:**
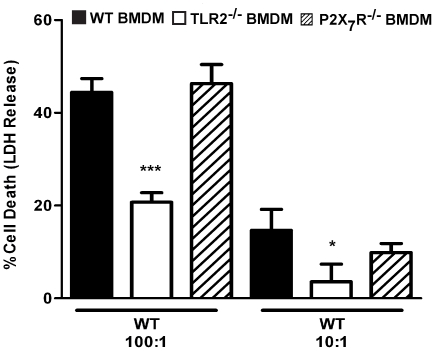
The P2X_7_ receptor is not required for *F. novicida*-dependent inflammasome activation. BMDMs from wild-type, TLR2^−/−^ and P2X_7_R^−/−^ mice were infected with wild-type *F. novicida* at the indicated MOIs. At 7 h post-infection, host cell death was measured by quantifying the amount of LDH released into the supernatant. Data are representative of three independent experiments. Error bars represent the standard deviation of triplicate samples. *** p≤0.0003, * p = 0.0319.

TLR signaling has previously been shown to be involved in inflammasome activation when triggered in conjunction with the P2X_7_ receptor (P2X_7_R) pathway, which leads to activation of the NLRP3 inflammasome [9]. We investigated whether the P2X_7_R pathway is involved in rapid inflammasome activation in response to *F. novicida* infection, which would potentially explain how TLR2 contributes to this process. We infected P2X_7_R^−/−^ macrophages with wild-type *F. novicida* and found that the levels of cell death (Figure 6) and IL-18 secretion (data not shown) were similar to the levels measured in infected wild-type macrophages at 7 h post-infection. In contrast, TLR2^−/−^ macrophages displayed defective responses. These findings demonstrate that *F. novicida* induces rapid inflammasome activation through a P2X_7_R-independent mechanism. This is in agreement with results from us and others demonstrating that *F. novicida*-induced inflammasome activation is independent of NLRP3 [9], [11], [43]. Therefore, the previously identified TLR/P2X_7_R/NLRP3 pathway cannot explain the role of TLR2 in rapid inflammasome activation by *F. novicida*.

### TLR2 plays a role in inflammasome activation in response to *Listeria*

Since we have shown that TLR2 is required for rapid inflammasome activation in response to *F. novicida*, we tested whether TLR2 plays a similar role in inflammasome activation during infection with another cytosolic bacterial pathogen known to activate the AIM2 inflammasome, *Listeria monocytogenes* [4]. We found that 15% of wild-type macrophages infected with *L. monocytogenes* at an MOI of 20∶1 underwent cell death at 1 h post-infection, while only 3% of TLR2^−/−^ macrophages died (Figure S6). This difference in cell death was also exhibited in macrophages infected at an MOI of 1∶1 at 5 h post-infection (data not shown). These results provide evidence demonstrating that TLR2 promotes inflammasome activation in response to multiple cytosolic bacterial pathogens.

### TLR2 is required for rapid *F. novicida*-induced inflammasome activation *in vivo*

**Figure 7:**
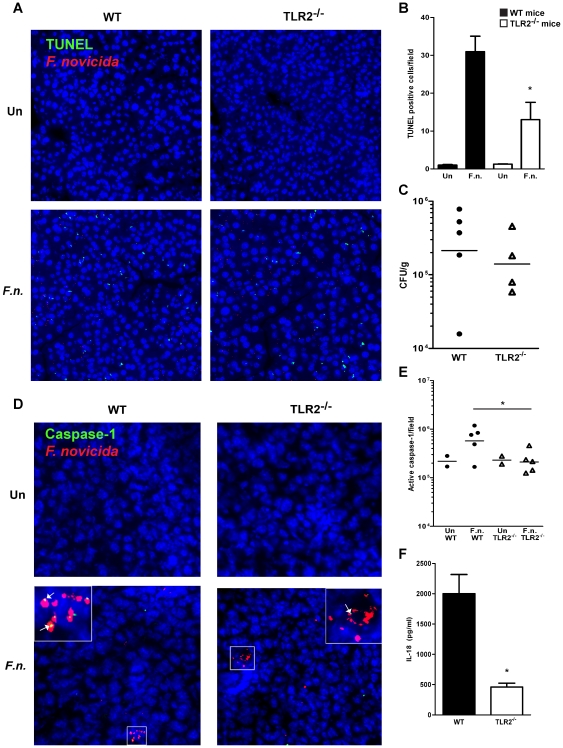
TLR2 plays a role in inflammasome activation *in vivo*. Wild-type and TLR2^−/−^ mice were left uninfected (Un) or infected intraperitoneally with 2.4×10^6^ CFU of wild-type *F. novicida* (*F.n*.) and livers were harvested at 4 h post-infection. (A) Cell death in the liver was visualized by TUNEL staining, and (B) the number of TUNEL positive cells per field was quantified. (C) The bacterial burden in the liver of infected mice was determined by counting colony-forming units. (D) Sections were stained for presence of active caspase-1 (p20) in the livers of uninfected and *F. novicida*-infected mice, and (E) the level of caspase-1 activation was quantified by measuring the total fluorescence intensity emitted by the FITC-labeled p20 subunit of capase-1. (F) The amount of IL-18 present in the serum of *F. novicida*-infected mice was measured by ELISA. Data are representative of three independent experiments. Error bars represent the standard deviation of triplicate samples. ** p = 0.0030, * p≤0.0370.

We have shown that TLR2 signaling is required for rapid inflammasome activation in response to *F. novicida* infection in macrophages. To determine whether TLR2 plays a similar role in inflammasome activation *in vivo*, we infected wild-type and TLR2^−/−^ mice intraperitoneally with 2×10^6^ CFU of wild-type *F. novicida* and assayed for inflammasome activation using several readouts. TUNEL staining was used to assess the amount of host cell death in the livers of infected mice. At 4 h post-infection, we observed increased TUNEL staining in the livers of wild-type mice in comparison to the livers of TLR2^−/−^ mice (Figures 7A, B). This response was independent of any differences in bacterial loads in infected livers since wild-type and TLR2^−/−^ mice harbored equivalent levels of bacteria at this early timepoint (Fig. 7C). This result is consistent with our finding that TLR2^−/−^ macrophages exhibited significantly less cell death in response to *F. novicida* infection *in vitro* (Figure 1).

To determine whether TLR2 contributes to caspase-1 activation *in vivo*, fluorescence microscopy was used to visualize the mature 20 kD subunit of active caspase-1 in the livers of infected wild-type and TLR2^−/−^ mice. We observed increased levels of active caspase-1 in the livers of wild-type mice compared to TLR2^−/−^ mice 4 h post-infection (Figures 7D, E). Previous studies have shown that the inflammasome can be visualized microscopically and that *F*. *novicida* colocalizes with this complex during macrophage infection [11], [12]. We observed *F*. *novicida* colocalizing with mature caspase-1 in the livers of infected mice, which may represent the first time that the active inflammasome has been visualized during bacterial infection *in vivo*.

Active caspase-1 cleaves pro-IL-18 resulting in its release into the extracellular environment. Therefore, we measured the amount of IL-18 present in the serum of infected mice. The concentration of IL-18 measured in the serum of TLR2^−/−^ mice was significantly reduced when compared to wild-type mice following infection (Figure 7F), and this was not due to differences in the expression of IL-18 mRNA (data not shown). Taken together, these results show that TLR2 is required for rapid inflammasome-dependent host cell death, caspase-1 activation and IL-18 production during *F. novicida* infection *in vivo*.

## Discussion

Upon infection of macrophages, *F*. *novicida* is initially recognized by TLR2 at the plasma membrane and in the phagosome, leading to the induction of a proinflammatory response dependent upon MyD88 and NF-κB [20], [37]. Subsequently, the bacteria escape the phagosome and reach the cytosol where they are recognized by the inflammasome, leading to death of the infected macrophage and the release of the proinflammatory cytokines IL-1β and IL-18 [13]. Here, we show that TLR2, in addition to inducing proinflammatory cytokine production through the canonical MyD88/NF-κB pathway, contributes to rapid inflammasome activation in response to *F. novicida* infection.

TLR2^−/−^ macrophages exhibited a delayed inflammasome response compared to wild-type macrophages, as indicated by delayed inflammasome assembly, levels of caspase-1 maturation, host cell death and IL-18 secretion (Figures 1, 2, 3). Interestingly, TLR2^−/−^ macrophages did undergo inflammasome-dependent cell death after *F. novicida* infection, in contrast to ASC^−/−^, caspase-1^−/−^and AIM2^−/−^ macrophages, which do not die up to 14 h post-infection ([11], [13] and data not shown). This suggests that TLR2 is not absolutely required for inflammasome activation, but instead contributes to rapid induction of inflammasome activity. Consistent with these *in vitro* findings, we observed decreased levels of mature caspase-1 and host cell death in the livers of *F. novicida*-infected TLR2^−/−^ mice compared to wild-type mice, as well as decreased levels of IL-18 in the serum (Figure 7). Recently, it was reported that highly virulent *F. tularensis* strains induced caspase-3-dependent cell death in mice [44]. It would be interesting to test whether TLR2 contributes to cell death during infection with these strains as well. Taken together, these findings clearly demonstrate that TLR2 plays an important role in promoting rapid inflammasome activation during *F. novicida* infection.

The role of TLR2 is not explained by differences in the expression levels of AIM2 inflammasome components since TLR2 signaling does not significantly alter the expression of AIM2 (Figure S4), ASC or caspase-1 (Figure 3). It is, however, possible that TLR2 signaling induces the expression of an as yet unidentified inflammasome component, explaining its effect on inflammasome activation. AIM2 expression is dependent on type I interferons (IFN) including IFN-β [45], which is also required for *F. novicida*-induced inflammasome activation [46]. Therefore, the role of TLR2 in rapid inflammasome assembly could be explained if TLR2 controlled IFN-β expression. In fact, Cole *et al* found that TLR2 regulates IFN-β expression in macrophages during *F. tularensis* Live Vaccine Strain (LVS) infection [37], [47]. However, we found equivalent levels of IFN-β mRNA in wild-type and TLR2^−/−^ macrophages following *F. novicida* infection (data not shown), in agreement with previous reports demonstrating that TLR adaptor proteins do not play a significant role in IFN-β expression during *F. novicida* infection [46]. In addition, our findings showed that AIM2 expression was not dependent on TLR2 signaling (Figure S4). Discrepancies between the present study and those of Cole *et al* could be due to differences in the kinetics of the induction of IFN-β in macrophages infected with *F. novicida*, which is more rapid than during *F. tularensis* LVS infection [37], [46]. This rapid induction of IFN-β by *F. novicida* may mask the potential contribution of TLR2 to its expression.

AIM2 inflammasome activation has previously been shown to occur in response to bacterial and viral infections [4], but can also be induced by transfection of double-stranded DNA into host cells, bypassing the need for TLRs [48], [49]. Our finding that TLR2 is not absolutely required for *F. novicida*-induced inflammasome activation is in agreement with the ability of the AIM2 inflammasome to be activated independently of TLRs. However, in the context of *F. novicida* infection, we find that TLR2 nonetheless promotes more rapid AIM2 inflammasome activation (Figure 1A). To the best of our knowledge, the contribution of TLR signaling in promoting rapid AIM2 inflammasome activation during infection with the intracellular bacterium *F. novicida* is novel.

TLR signaling has been linked to NLRP3 inflammasome activation when macrophages are costimulated with TLR ligands and extracellular ATP, which activates the P2X_7_R pathway [9]. However, we find that the P2X_7_R pathway is not required for rapid *F. novicida*-induced inflammasome activation (Figure 6), and we and others have previously shown that this process occurs independently of NLRP3 [9], [11], [43]. This demonstrates that the role of TLR2 in promoting rapid inflammasome activation cannot be explained by cooperation with the P2X_7_R/NLRP3 pathway. However, TLR2 signaling through NF-κB induces the expression of a multitude of proteins involved in host defense, including antimicrobial peptides [50]. It is possible that TLR2 indirectly promotes inflammasome activation by increasing the expression of NF-κB-dependent antimicrobial peptides, which damage the bacteria, leading to increased release of bacterial DNA, the activator of the AIM2 inflammasome. Whether antimicrobial peptides or other defenses contribute to release of bacterial DNA and inflammasome activation is an important future question to answer.

Additionally, we show that TLR2 contributes to inflammasome activation in response to infection by *L*. *monocytogenes*, a cytosolic bacterium that is also recognized by AIM2 (Figure S6). In a previous study by Ozoren *et al*, TLR2 was shown to play no role in inflammasome activation in thioglycollate-elicited peritoneal macrophages infected with *L*. *monocytogenes* at an MOI of 50∶1 [51]. The high multiplicity of infection used by the authors of that study, which was higher than those used in the present study, could have forced the infection to proceed more rapidly, thereby masking the role of TLR2 in rapid inflammasome activation. In fact, we found that bone marrow-derived macrophages infected with *L*. *monocytogenes* at MOIs of 50∶1 and 100∶1 did not require TLR2 for inflammasome activation (data not shown). It would be interesting to test whether TLRs play a role in promoting more rapid AIM2 inflammasome activation during infections with other pathogens that activate AIM2 such as vaccinia virus and murine cytomegalovirus [4].

Our results suggest that TLR2 and the AIM2 inflammasome provide an integrated, multi-tiered recognition and defense system against intracellular bacteria. TLR2 recognizes the presence of both pathogenic and nonpathogenic bacteria at the plasma membrane and in the phagosome of macrophages, whereas the AIM2 inflammasome recognizes pathogenic bacteria that escape the phagosome and reach the cytosol. The initial induction of the TLR2 signaling pathway promotes more rapid AIM2 inflammasome activation only in the event that bacteria reach the cytosol. This enables the macrophage to elicit an innate response based on the level of the threat imposed by invading bacteria. Inflammasome-mediated host cell death is a protective host response that removes the bacterium's replicative niche, but also comes with a significant cost since it depletes macrophages as well. Therefore, this response is tightly regulated and only induced when the macrophage encounters a heightened level of danger that is triggered when bacteria reach the cytosol. Taken together, we propose a model in which macrophages integrate signals from the spatiotemporally separated TLR2 signaling pathway and AIM2 inflammasome complex during infection in order to mount an appropriate innate immune response against invading bacteria.

## Supporting Information

Figure S1 **Phagosomal escape and intracellular replication of** ***F. novicida*** **in BMDM.** (A) Macrophages were infected with *F. novicida* at an MOI of 100∶1 and stained as described in the *Materials and Methods*. At 30 and 60 minutes postinfection, the number of bacteria colocalized with LAMP-1-containig vacuoles was enumerated. At least 100 bacteria were analyzed per sample. (B) Macrophages were infected with *F. novicida* at an MOI of 20∶1. At 5 h post-infection, before any visible cell death occurred, macrophage lysates were plated and grown overnight. To determine the bacterial load, colony forming units (CFU) were counted.
(TIF)

Figure S2 **TLR2 is not required for IL-18 expression.** BMDM from wild-type and TLR2^−/−^mice were left uninfected (Un) or infected with *F. novicida* (F.n.) at an MOI of 100∶1. At 5.5 h post-infection, macrophages were lysed. (A) RNA was harvested and quantitative real-time PCR was used to determine the level of IL-18 expression, which is represented relative to the expression of the housekeeping gene β-actin. (B) The total amount of IL-18 present in each lysate was measured by ELISA. Data are representative of three independent experiments.
(TIF)

Figure S3 **TLR2** ^−/−^ **macrophages exhibit delayed caspase-1 activation in response to** ***F. novicida*** **infection.** WT and TLR2^−/−^ BMDM were infected with wild-type and the *mglA* mutant strain of *F. novicida* at an MOI of 100∶1 for 7.5 h. Macrophage lysates were collected, and the presence of procaspase-1, mature caspase-1 (p20 subunit) and β-actin was visualized by western blot.
(TIF)

Figure S4 **TLR2 is not required for AIM2 expression.** BMDM from wild-type and TLR2^−/−^ mice were left uninfected (Un) or infected with *F. novicida* (F.n.) at an MOI of 100∶1. At 5.5 h post-infection, macrophages were lysed and the total RNA was harvested. Quantitative real-time PCR was used to determine the level of AIM2 expression, which is represented relative to the expression of the housekeeping gene β-actin. qRT-PCR analysis was used to quantify AIM2 expression because we were unable to detect the protein by western blot in macrophages that were not pre-treated with IFN-β. Data are representative of three independent experiments. Bars represent the geometric mean for each group.
(TIF)

Figure S5 **TLR2 signaling is not sufficient to induce cell death.** BMDMs from wild-type and TLR2^−/−^ mice were either left untreated (no Tx), stimulated with Pam_3_CSK_4_ (BLP), heat-killed wild-type *F. novicida* (HK F.n.), or Ultrapure *E. coli* LPS or infected with the *mglA* mutant strain of *F. novicida* (F.n. MglA) at an MOI of 100∶1. At 7 h, (A) the concentration of IL-6 in the supernatants was measured by ELISA, and (B) cell death was measured by quantifying the amount of LDH released into the supernatants. Data are representative of three independent experiments. Error bars represent the standard deviation of triplicate samples. ***p values <0.0008.
(TIF)

Figure S6 **TLR2 plays a role in inflammasome activation during** ***Listeria*** **infection.** Wild-type and TLR2^−/−^ BMDM were infected with *L. monocytogenes* EGD-e at an MOI of 20∶1 for 1 h. At the indicated timepoints, cell death was measured by quantifying the amount of LDH released into the supernatants. Data are representative of three independent experiments. **p = 0.0021.
(TIF)
