# Supplementary figures and images for: TLR2 Signaling Contributes to Rapid Inflammasome Activation during F. novicida Infection
Source: PLoS One. 2011 Jun 16;6(6):e20609. doi: 10.1371/journal.pone.0020609 (PMC3116832; doi:10.1371/journal.pone.0020609)

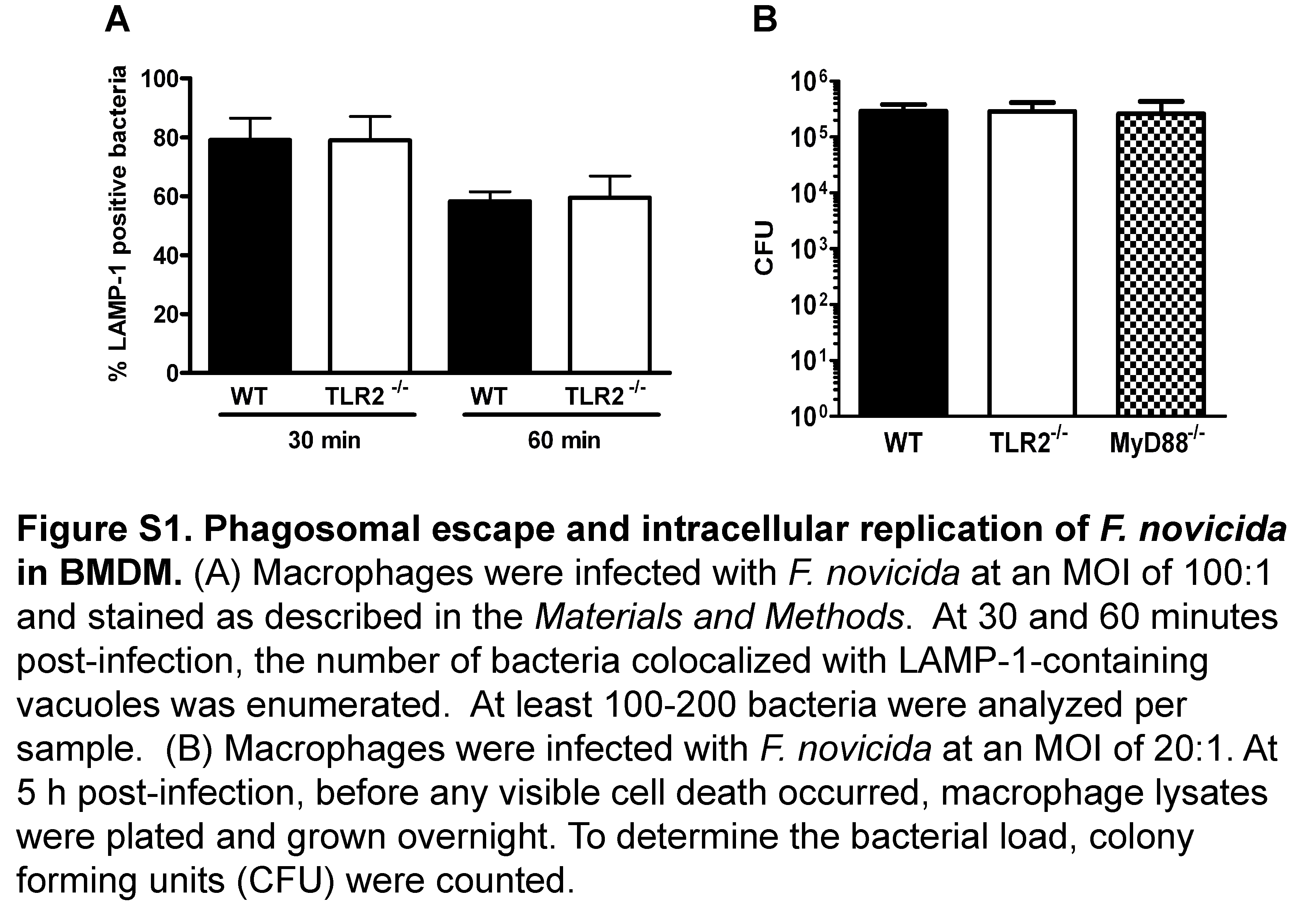

Supplement: Figure S1 — Phagosomal escape and intracellular replication of F. novicida in BMDM. (A) Macrophages were infected with F. novicida at an MOI of 100∶1 and stained as described in the Materials and Methods . At 30 and 60 minutes postinfection, the number of bacteria colocalized with LAMP-1-containig vacuoles was enumerated. At least 100 bacteria were analyzed per sample. (B) Macrophages were infected with F. novicida at an MOI of 20∶1. At 5 h post-infection, before any visible cell death occurred, macrophage lysates were plated and grown overnight. To determine the bacterial load, colony forming units (CFU) were counted. (TIF) [file pone.0020609.s001.tif]

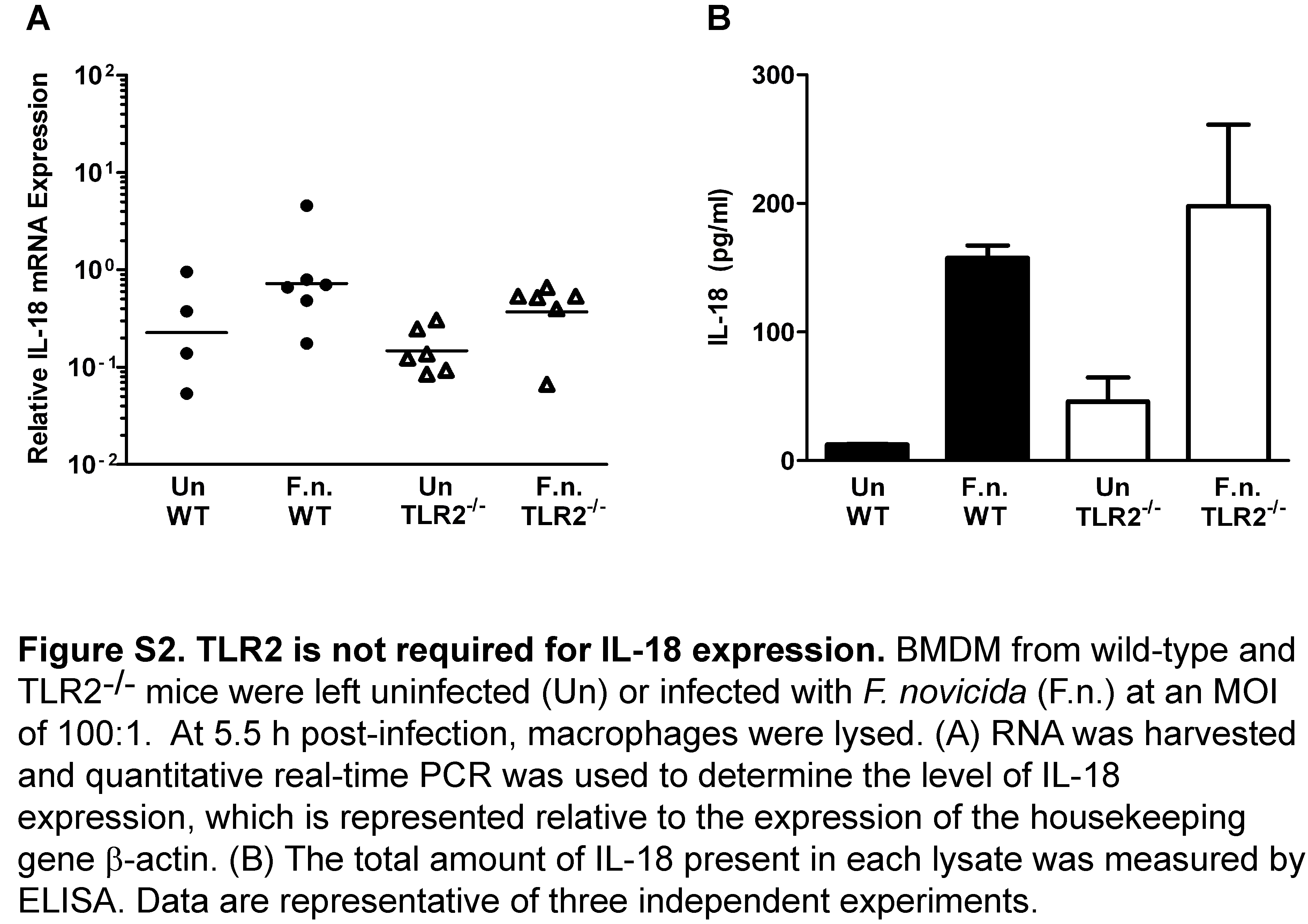

Supplement: Figure S2 — TLR2 is not required for IL-18 expression. BMDM from wild-type and TLR2−/−mice were left uninfected (Un) or infected with F. novicida (F.n.) at an MOI of 100∶1. At 5.5 h post-infection, macrophages were lysed. (A) RNA was harvested and quantitative real-time PCR was used to determine the level of IL-18 expression, which is represented relative to the expression of the housekeeping gene β-actin. (B) The total amount of IL-18 present in each lysate was measured by ELISA. Data are representative of three independent experiments. (TIF) [file pone.0020609.s002.tif]

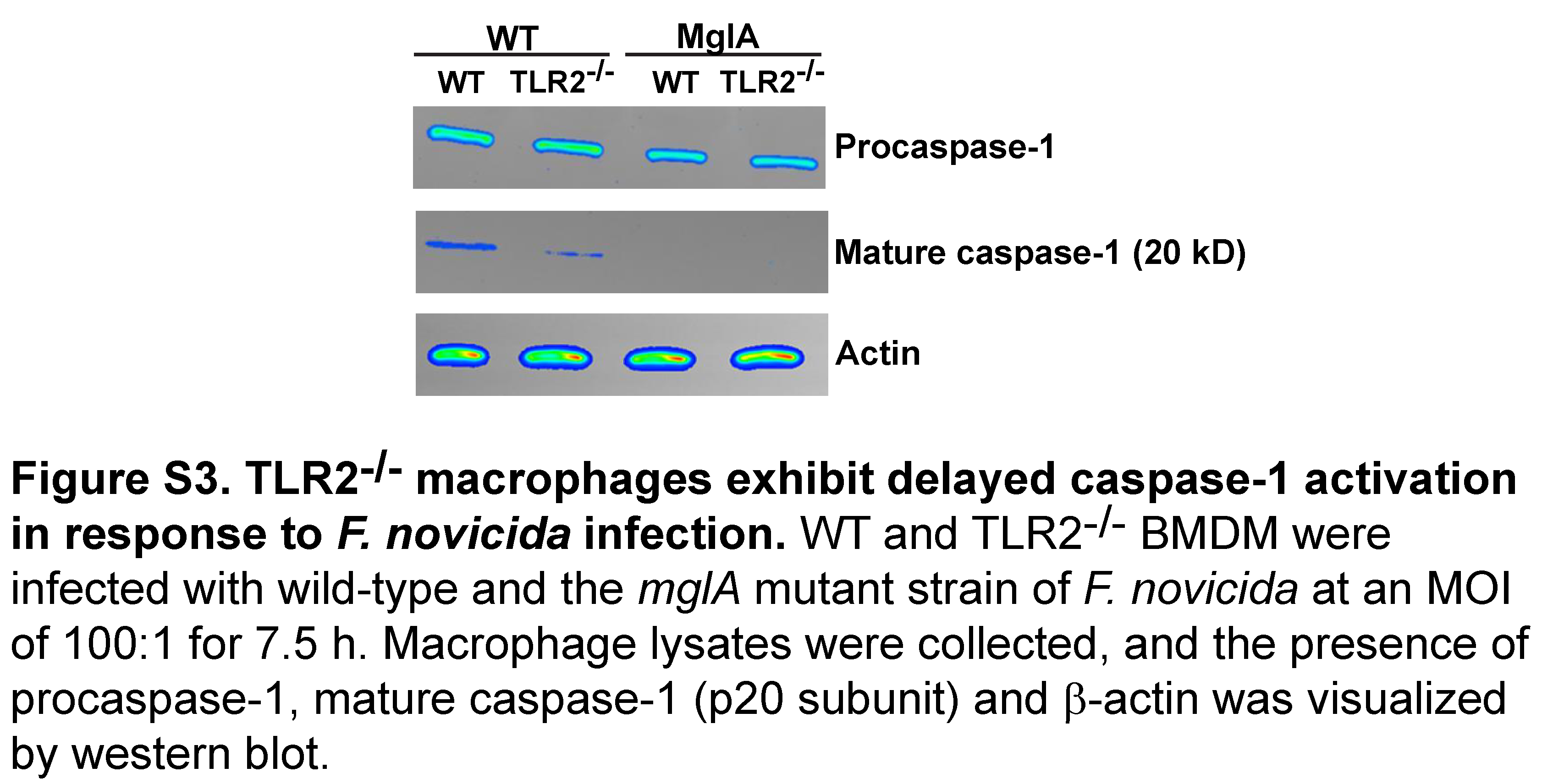

Supplement: Figure S3 — TLR2 −/− macrophages exhibit delayed caspase-1 activation in response to F. novicida infection. WT and TLR2−/− BMDM were infected with wild-type and the mglA mutant strain of F. novicida at an MOI of 100∶1 for 7.5 h. Macrophage lysates were collected, and the presence of procaspase-1, mature caspase-1 (p20 subunit) and β-actin was visualized by western blot. (TIF) [file pone.0020609.s003.tif]

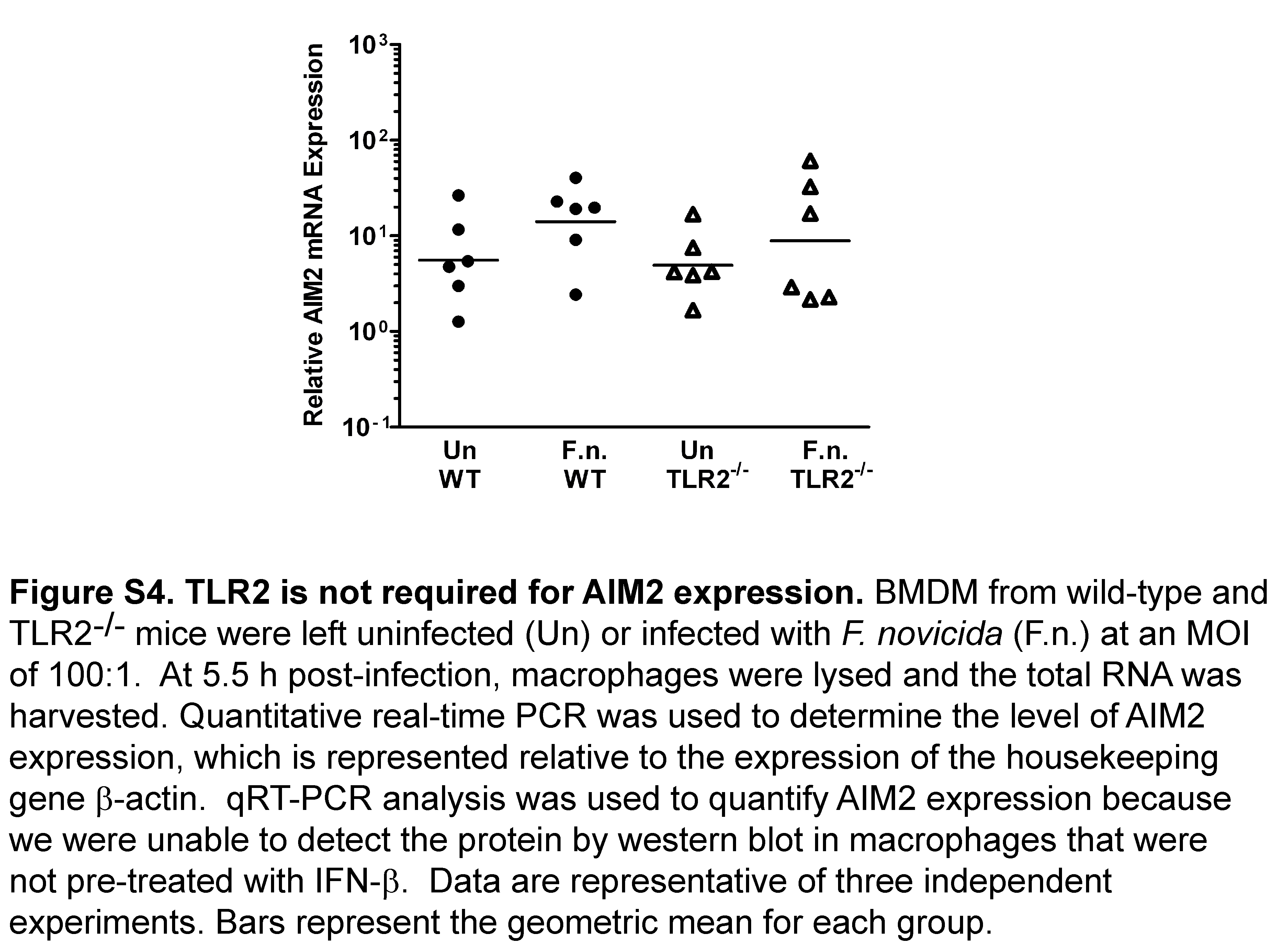

Supplement: Figure S4 — TLR2 is not required for AIM2 expression. BMDM from wild-type and TLR2−/− mice were left uninfected (Un) or infected with F. novicida (F.n.) at an MOI of 100∶1. At 5.5 h post-infection, macrophages were lysed and the total RNA was harvested. Quantitative real-time PCR was used to determine the level of AIM2 expression, which is represented relative to the expression of the housekeeping gene β-actin. qRT-PCR analysis was used to quantify AIM2 expression because we were unable to detect the protein by western blot in macrophages that were not pre-treated with IFN-β. Data are representative of three independent experiments. Bars represent the geometric mean for each group. (TIF) [file pone.0020609.s004.tif]

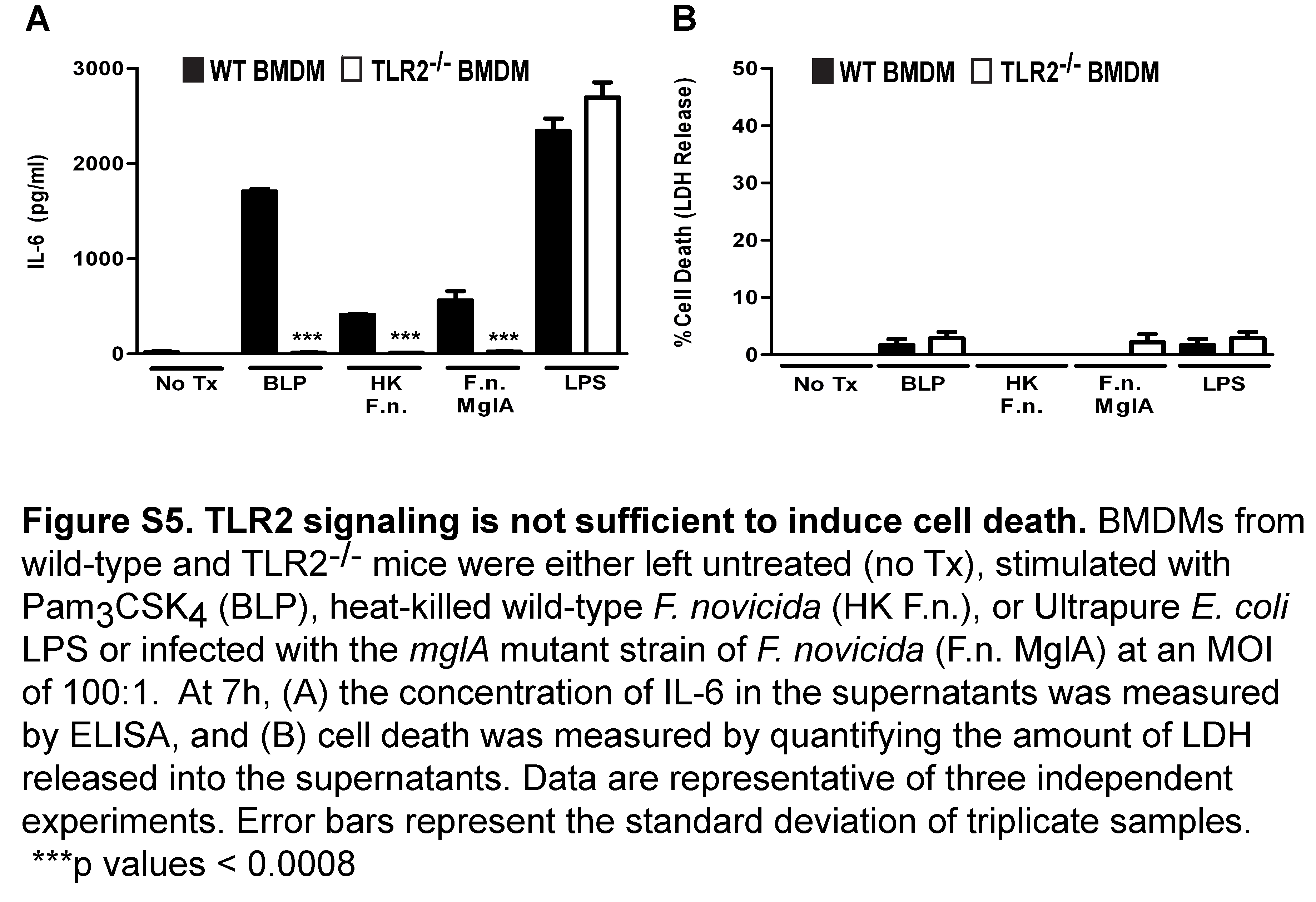

Supplement: Figure S5 — TLR2 signaling is not sufficient to induce cell death. BMDMs from wild-type and TLR2−/− mice were either left untreated (no Tx), stimulated with Pam3CSK4 (BLP), heat-killed wild-type F. novicida (HK F.n.), or Ultrapure E. coli LPS or infected with the mglA mutant strain of F. novicida (F.n. MglA) at an MOI of 100∶1. At 7 h, (A) the concentration of IL-6 in the supernatants was measured by ELISA, and (B) cell death was measured by quantifying the amount of LDH released into the supernatants. Data are representative of three independent experiments. Error bars represent the standard deviation of triplicate samples. ***p values <0.0008. (TIF) [file pone.0020609.s005.tif]

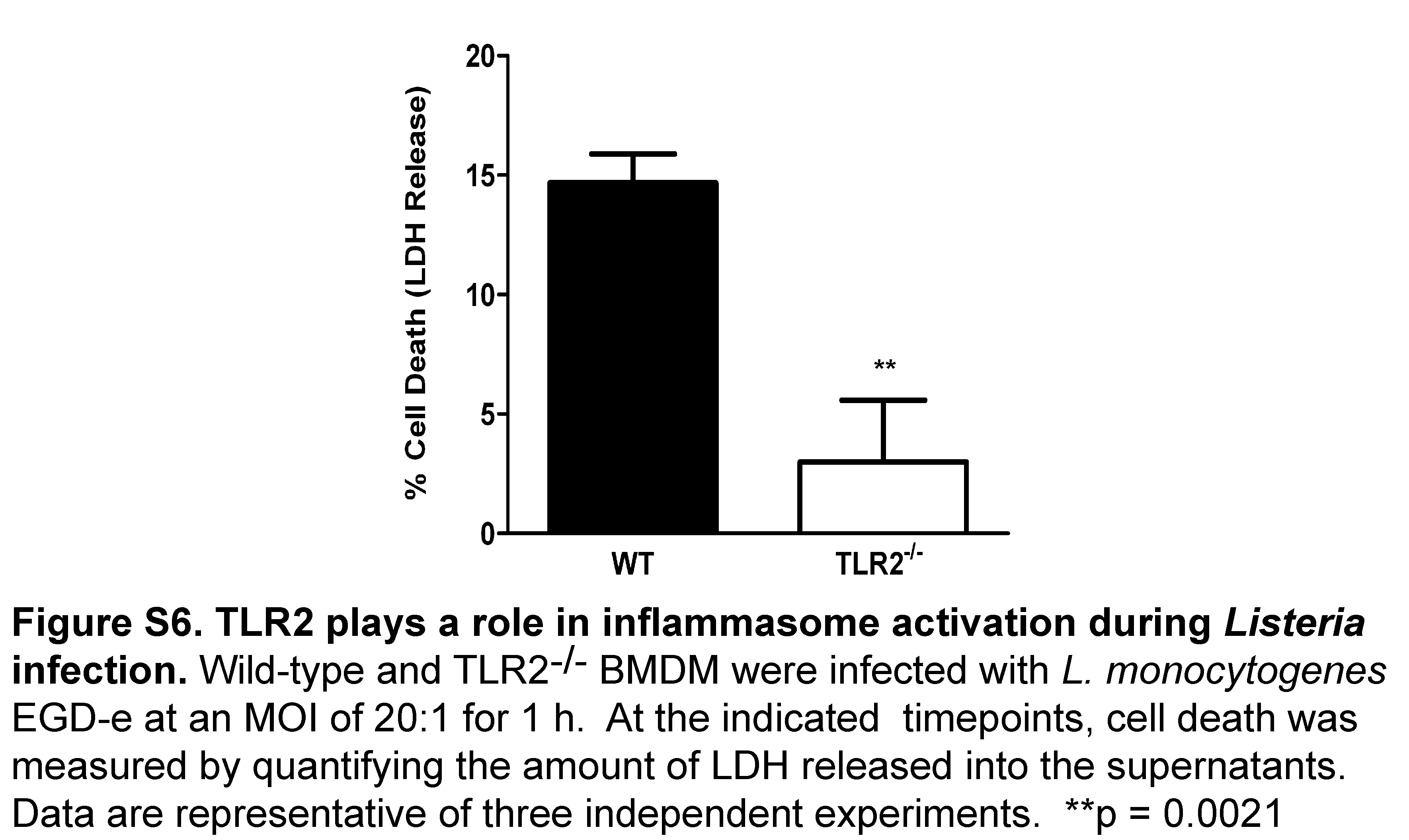

Supplement: Figure S6 — TLR2 plays a role in inflammasome activation during Listeria infection. Wild-type and TLR2−/− BMDM were infected with L. monocytogenes EGD-e at an MOI of 20∶1 for 1 h. At the indicated timepoints, cell death was measured by quantifying the amount of LDH released into the supernatants. Data are representative of three independent experiments. **p = 0.0021. (TIF) [file pone.0020609.s006.tif]
